# Underlying factors influencing job satisfaction and stress among TN-visa workers in the U.S. swine industry

**DOI:** 10.1016/j.onehlt.2026.101500

**Published:** 2026-06-27

**Authors:** Magnus R. Campler, Andréia G. Arruda, Kara Flaherty, Talita Resende, Isaiah Franco, Douglas Jackson-Smith, Timothy J. Safranski, Magdiel Lopez-Soriano

**Affiliations:** a College of Veterinary Medicine, Department of Veterinary Preventive Medicine, The Ohio State University, Columbus, OH, USA; b School of Environment and Natural Resources, The Ohio State University, Wooster, OH, USA; c Extension Department- Swine, University of Missouri, Columbia, MO, USA

**Keywords:** Job satisfaction, Farm workers, Employee turnover, Workplace stress, Immigrants

## Abstract

The United States (U.S.) swine industry has increasingly relied on North American Free Trade Agreement (NAFTA) visa program workers (TN-Visa) to combat labor shortages. However, high turnover rates are also reported among TN-visa workers, but the underlying causes are mainly unknown. The study objectives were to 1) describe TN-Visa participants' demographics and 2) explore underlying factors for job satisfaction, stress levels and the likelihood of changing employers for TN-Visa workers. A survey consisting of a mix of demographic, ranking, and open-ended questions was created and translated to Spanish. The survey was distributed online (*N* = 50) and in-person (*N* = 211) to TN-Visa workers in Iowa, Missouri, Indiana, Minnesota, Ohio, and North Carolina. Mixed multivariable logistic regression models were used to identify predictors of job satisfaction, stress levels, and job change intent. High stress levels were associated with lower job satisfaction. Respondents with over 72 months of TN-visa program tenure were 80% less likely to experience a high level of job satisfaction compared to those with shorter experience, but older TN-visa workers showed higher job satisfaction compared to younger workers. High job satisfaction or moderately sized farm employment decreased the likelihood of considering changing their employer. Previous animal husbandry experiences doubled the likelihood of considering changing their employer compared to workers with no previous animal husbandry experience. These findings underscore the relevance of a One Health approach, as worker wellbeing and retention are closely linked to animal health and welfare; and overall farm sustainability, with improved job satisfaction and reduced stress potentially benefiting both human and animal health outcomes. This study is one of few to present underlying factors for self-reported job satisfaction, stress, and changing employment for TN-visa workers and provides the U.S swine industry stakeholders with important insights into TN-visa working conditions.

## Highlights

• Long-term (>72 months) TN-visa participation increased odds of job dissatisfaction.
• Previous animal husbandry experience increased odds of potential turnover.
• Employment on medium-sized farms decreased odds of potential turnover.

## 1 Introduction

Over the last 40 years, farmers in the United States (U.S.) have become increasingly dependent on immigrant farm labor to meet their needs. These vertically integrated food systems have increasingly shifted to rely on formal temporary guest worker programs [1], [2], [3]. This phenomenon has led commercial farms to increasingly utilizethe formerly called North American Free Trade Agreement (NAFTA) TN-Visa program to fill their needs [4]. The TN-Visa program allows skilled Canadian and Mexican workers with bachelor's degrees to work and live in the U.S. for up to three years while being eligible to re-apply at the end of each term [5]. Labor shortages have been a particular problem in the U.S. swine industry, where vacancies have increased over the last decade [6] with turnover rates reported between 20 and 35% for U.S. swine farms [7]. To fill vacancies, TN workers have become a particularly important source of labor for the U.S. swine industry, with TN workers predominantly sourced from Mexico with the prerequisite of holding a minimum of a bachelor's degree in fields such as animal science, agriculture, or veterinary medicine [8], [9]. Despite the industry paradigm shift in labor recruitment since the elimination of the TN visa cap in 2004 [10], the U.S. swine industry still suffer from high turnover rates for both domestic and TN-visa workers [8], [11]. The TN-visa is conditional to employment under little government oversight, with reports of less-than-ideal employer practices with conditions including low pay, and substandard working conditions and/or withheld benefits, possibly adding to increased turnover [12], [13]. To date, research on working conditions, stress and job satisfaction of TN-visa workers in the U.S swine industry is very limited.

To date, research on TN-visa worker working conditions and job satisfaction in the U.S swine industry is limited. To fill this gap, our team surveyed 261 TN-visa workers on U.S. swine farms with the main objective of exploring underlying factors associated with job satisfaction, stress levels, and the likelihood of changing employer for TN-visa workers.

## 2 Material and methods

### 2.1 Study approval and data management

This research was evaluated and approved by the Missouri University Institutional Review Board (IRB, protocol #2101146-MU) and classified as exempt by The Ohio State University's IRB (protocol #2024E0680). After survey responses were collected, any identifying data were anonymized and transcribed into Excel® (Microsoft Corp., Redmond, WA) and stored internally within an institutional two-factor authenticated cloud-based resource. All participants needed to consent to participate before beginning the survey.

### 2.2 Participant recruitment

Large swine operations and regional swine industry stakeholders were contacted to invite TN-visa workers to attend in-person meetings to complete a 45-min survey. All participants were informed about the project and provided with a consent form to participate in the survey. A $25 gift card would be provided to workers completing their survey in person. The link to the survey was also uploaded in Qualtrics® and published on LinkedIn (Sunnyvale, California, U.S.) and several TN-visa groups on Meta® (Menlo Park, California, U.S.) to reach TN-visa workers outside of the initial target area. Incentives were not offered to online participants. All in-person participants were asked not to participate in the online survey to avoid double participation.

### 2.3 Survey design

The survey was designed based on similar survey study designs targeting farm employees [14], [15] and in collaboration with the Assessment Resource Center (ARC) at the University of Missouri. The ARC involvement also ensured that the collected data and related activities complied with the established Human Research Protection Program.

Thirty-six questions were designed to collect data on six topics: 1) current farm characteristics, 2) respondent demographics 3) self-reported personal and professional goals, 4) access to benefits and training opportunities, 5) levels of stress and job satisfaction, and 6) stated desire to leave their current employer. Per the scope of this study's outcomes, partial respondent demographics and four questions related to the topics of benefits, job stress and job satisfaction, and stated desire to leave their current employer and their associated open-ended answers were investigated. The survey included a mix of demographic questions, closed question formats (yes/no, check all-that-apply, and 5-point rating scales), as well as a few open-ended questions.

The final survey was translated into Spanish by a native Spanish speaking team member. To ensure accuracy and clarity, bilingual human resources professionals and technical swine training personnel from private swine companies reviewed the survey's translated version before it was sent to the applicable population. This review and approval process helped standardize terminology and increase the overall comprehension level of the target group. Although formal back-translation of the instrument was not conducted, the multi-reviewer process provided both literacy-specific and linguistic validation for the survey. A survey completion criterion was considered at 80%. The full survey in both English and Spanish can be found here [16].

### 2.4 Data management and analysis

For data analysis purposes, some categorization was conducted considering sample size for each level, and in an attempt to keep as many categories as possible for richness of information; survey-type (online, in-person), TN-visa program (≤36 months, 37–72 months, and >72 months), swine herd size (0–2500, 2501–5000, and ≥5001), job position (farm manager, production manager trainee, team lead, and hourly employee), age (18–34, 35–44, and 45–64) and educational level (bachelor's degree or higher degree), English proficiency (very little, little, some, most, or everything), training (weekly, monthly, yearly, never), and previous animal husbandry experience (yes/no), and investigated as potential predictors.

For open-ended questions a summative content analysis approach was conducted to establish categories for answers describing similar topics using key words, meanings, and concepts [17]. Three experienced co-authors independently reviewed and categorized each open-ended response after which each response was revisited, reviewed among the three reviewers until a consensus was reached for the appropriateness of each response's belonging to a category and overarching theme. The frequency of answers belonging to each theme of the open-ended answers is reported descriptively.

Three outcome variables; *job satisfaction* (very unsatisfied, somewhat unsatisfied, neither satisfied nor unsatisfied, somewhat satisfied, or very satisfied), *stress level* (not stressful at all, not very stressful, neither stressful or not stressful, a little stressful, or very stressful), and *thinking about changing employer* (yes/no) were used as dependent variables for multivariable modeling. A Spearman Rank's correlation was made to investigate possible multicollinearity between potential explanatory variables using a cut-off coefficient of ≥0.6. Only pairs of explanatory variables below this cutoff were included in the models reported below.

Multivariable mixed effects ordinal logistic regression models were used to identify predictors of job satisfaction and worker stress level, while a multivariable mixed effects logistic regression model was used to explore predictors of the dichotomous dependent variable (thinking about changing employer). Respondent's home state was used as a random effect. To avoid over-fitting, univariable models were created for each explanatory variable of interest with a conservative cut-off value of *P* < 0.2 used to gauge inclusion of variables into full models. A backwards stepwise model building criteria was then applied. Any confounders were determined by >20% coefficient change during the removal of a non-significant variable. All preferred models resulted in reduced Akaike's Information Criterion (AIC) and the Bayesian Information Criterion (BIC). All analyses were conducted using Stata 18 (College Station, Texas). Statistical significance within the final model was declared at *P* *<* 0.05.

## 3 Results

### 3.1 Respondent characteristics

After screening and omission of incomplete surveys a total of 50 online responses and 211 in-person responses were obtained. Online respondents were distributed across 25 companies while in-person respondents were distributed across 10 companies. In total, taking into account the overlap between online and in-person responses, 30 companies were represented in the study. A majority were between the ages of 18–34 (60.8%), while 28.8% were 35–44, and 10.4% between the ages of 45–65. Nearly two-thirds identified as male (63.2%) and 36.0% as female, while 0.8% preferred not to say. Among respondents, 45.6% had less than 36 months of TN-visa experience, while 40.6% and 13.8% of the respondents had 36–72, or more than 72 months experience, respectively.

Approximately two-thirds (68.5%) of the respondents had previous animal husbandry experience but only four (2.3%) had previous swine farm experience. For respondents lacking previous animal husbandry experience, most had previous experience working with crops (72.5%) or greenhouse sectors (40.0%), followed by small veterinary clinics (13.8%), agricultural sales (12.5%), packing plants (12.5%), governmental jobs (8.8%) or other types of employment (15.0%).

Most TN-visa workers in this study worked on larger farms with 18.2%, 39.3% and 42.4% working on farms with <2500, 2501-5000, and > 5000 pigs, respectively. The majority were employed as hourly employees (73.5%) followed by team lead (14.2%), production manager trainees (5.8%), farm managers (5.0%), or other specialized positions (1.5%).

The self-reported English proficiency was low with approximately two-thirds (68.9%) limited to a few English words or phrases, while approximately a third (31.1%) were capable of both understanding and communicating well in English. The full breakdown on the levels of English proficiency can be found in Supplementary Table 1.

### 3.2 Job satisfaction

**Fig. 1:**
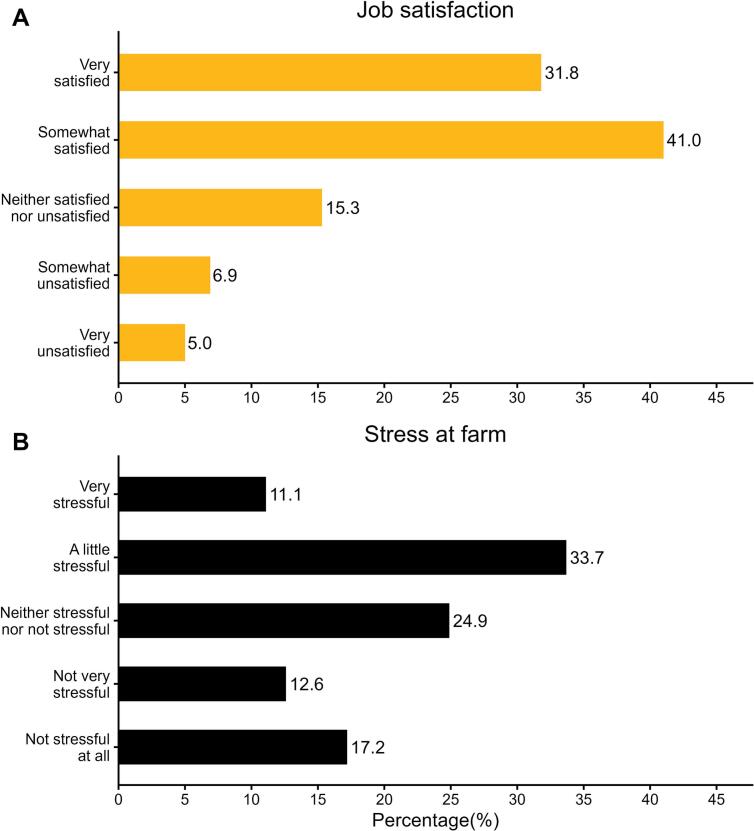
Distribution (%) of self-reported job satisfaction (A) and stress levels (B) among TN-visa workers.

**Fig. 2:**
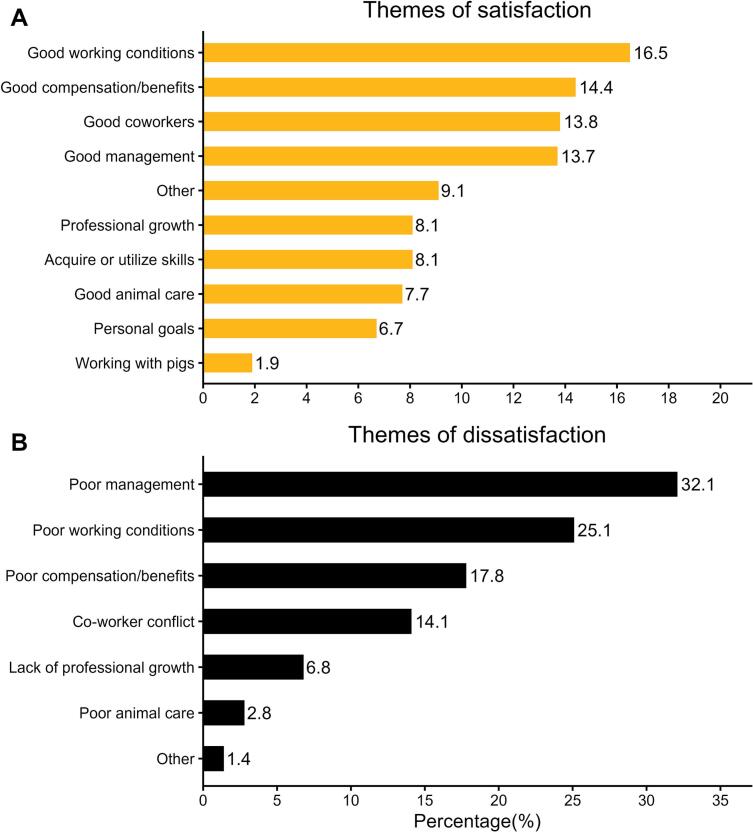
Content analysis of optional open-ended question for satisfied (A) and dissatisfied (B) job conditions for TN-visa survey respondents in the U.S swine industry. Results are presented as the percentage (%) of answers belonging to a specific theme.

Most of the TN-visa workers (72.8%) in our sample were somewhat satisfied or very satisfied with their current position (Fig. 1). A content analysis based on open-ended answers is provided in Fig. 2.

Some respondents that felt neither satisfied nor dissatisfied often described both pros and cons of their current workplace. In total, 26 satisfied answers and 31 dissatisfied answers were collected. A content analysis of the comments showed that the themes around positive answers were mainly tied to salary and benefits (52.0%) and professional growth (28.0%) while a few respondents appreciated the current work environment (8.0%), the job opportunity (4.0%), the teamwork (4.0%), and the company management (4.0%). On the dissatisfied side, the main themes were associated with poor management (34.5%), heavy workload (20.7%), lack of appropriate salary and benefits (17.2%), and poor working environment (13.7%). Some respondents mentioned inequality between domestic and TN-visa workers (10.3%) and one respondent stated that they felt neither satisfied nor unsatisfied because the farm was closing (3.6%).

### 3.3 Job benefits

Respondents revealed great variation between the benefits provided to TN-visa workers in our sample (Table 1) and between companies with more than five respondents (Supplementary Table 2). Almost all TN-visa workers (97.7%) were given paid time off (PTO), and the majority had access to health insurance care (79.7%), retirement plans (63.2%), or production bonuses (59.8%). Less than half (47.1%) indicated they were given referral bonuses and about a quarter (27.2%) were given the opportunity to attend special training courses or conferences (Table 1). Paid vacation and access to health insurance were considered very important by approximately 80% of the respondents. Around two thirds of the respondents considered production bonuses, retirement plans, or special training/conferences to be very important while 46.3% considered referral bonuses to be very important. For respondents lacking job benefits, access to insurance and retirement plans ranked highest followed by production bonuses, PTO, and special training/conferences (Table 1).

### 3.4 Stress factors

Approximately one in nine (11.1%) perceived their job as ‘very stressful’, with another 33.9% indicating their job was ‘a little stressful’. A quarter of the respondents found that their current position was neither stressful or not stressful (25.0%) while 12.7% perceived their position as not very stressful and 17.3% did not find their current position stressful at all. A content analysis based on open-ended answers for respondents provided self-described reasons for higher stress levels to be predominantly caused by poor management (19.5%) and workload (18.7%) followed by co-worker conflicts (13.8%) and workload inequality (12.2%). Tough production goals (9.8%), poor working conditions (5.7%), poor communication (5.7%) and work monotony (3.3%) were other factors for job stress. Individual non-thematic answers were categorized as ‘other’ (11.4%).

**Table 1:** The proportion (%) of TN-visa workers (*N* = 261) receiving specific job benefits and their self-reported importance ranking.

Provided job benefits^a^	N	%
Paid vacation (PTO)	255	97.7
Health insurance	208	79.7
Retirement plan	163	63.2
Production bonus	156	59.8
Referral bonus	123	47.1
Special training/conference	71	27.2


	Very important	Important	Slightly important	Not important
Importance of provided job benefits				
Paid vacation (PTO) (*N* = 251)	79.3	19.1	0.8	0.8
Health insurance (*N* = 207)	78.7	16.4	3.9	1.0
Retirement plan (*N* = 163)	64.4	26.4	8.0	1.2
Production bonus (*N* = 155)	68.4	25.2	4.5	1.9
Referral bonus (*N* = 119)	46.2	33.6	10.1	10.1
Special training/conference (*N* = 70)	60.0	28.6	7.1	4.3

Importance of wanted job benefits^b^				
Paid vacation (PTO) (*N* = 4)	75.0	25.0	0	0
Health insurance (*N* = 44)	93.2	6.8	0	0
Retirement plan (*N* = 73)	84.9	12.3	1.4	1.4
Production bonus (*N* = 84)	76.2	21.4	2.3	0
Referral bonus (*N* = 58)	69.0	27.6	1.7	1.7
Special training/conference (*N* = 130)	72.3	23.9	1.5	2.3

a Not all respondents answered all of the benefit importance questions.

b Only include respondents that did not have the particular benefit.

### 3.5 Interest in seeking a new employer

When asked if they considered changing employer, 40.2% answered ‘yes’, 56.3% answered ‘no’, while 3.5% did not answer. The most common reasons to consider changing their employer were due to poor compensation and benefits (36.7%), followed by poor management (30.0%) and lack of training or professional growth (16.7%). The remaining causes mentioned were poor working conditions (6.7%), relocating (5.0%), co-worker conflict (3.2%), and conflict of interest (1.7%).

### 3.6 Model results

The Spearman Rank found correlations ρ ≥ 0.6 (ρ_range_ 0.68–0.80) between the language proficiency questions warranting variable omission due to multicollinearity. Thus, for modeling purposes, comprehension when spoken to was retained as most farm directions and daily communication most likely would occur in English.

The final multivariable mixed effects ordered logistic regression for ‘job satisfaction’ showed that in-person survey respondents had close to three-fold higher odds of experiencing a higher level of job satisfaction compared to online survey respondents and that respondents between the age of 35–44 and 45–64 had approximately two-fold and five-fold higher odds of having better job satisfaction compared to younger respondents, respectively (Table 2). Respondents with more than 72-months in the TN-visa program were less likely to experience a higher level of job satisfaction compared to respondents with less than 36 months of TN-visa program experience and respondents holding a production manager trainee position or hourly position were less likely to experience a higher level of job satisfaction compared to respondents holding a farm manager position (Table 2). Finally, respondents with neutral, little, or very high stress levels had lower odds of experiencing a higher level of job satisfaction compared to respondents that were not stressed at all (Table 2).

**Table 2:** Results from final multivariable mixed effects ordered logistic regression model investigating predictors for job satisfaction in U.S TN-visa workers^c^.

Variable	Categories	Odds ratio (SE)	95% CI	*P*-value
Survey type	Online	*Referent*		
	In person	2.76 (1.03)	1.33–5.74	*0.007*
Age	18–34	*Referent*		
	35–44	2.14 (0.68)	1.15–4.00	*0.016*
	45–65	5.42 (2.71)	2.03–14.4	*0.001*
Time in the TN-visa program (months)	≤36	*Referent*		
	37–72	0.98 (0.28)	0.56–1.72	0.96
	>72	0.22 (0.09)	0.09–0.51	*<0.001*
Position	Farm manager	*Referent*		
	Production manager trainee	0.15 (0.13)	0.03–0.78	*0.024*
	Team lead	0.38 (0.28)	0.09–1.64	0.19
	Hourly employee	0.27 (0.18)	0.07–1.03	0.055
Training	Weekly	*Referent*		
	Monthly	0.48 (0.28)	0.16–1.48	0.20
	Yearly	0.82 (0.56)	0.21–3.15	0.77
	Never	0.38 (0.23)	0.12–1.26	0.11
Stress level	Not stressful at all	*Referent*		
	Somewhat stressful	0.45 (0.25)	0.15–1.31	0.14
	Neither stressful nor not stressful	0.35 (0.15)	0.15–0.82	*0.016*
	A little stressful	0.36 (0.15)	0.16–0.81	*0.014*
	Very stressful	0.05 (0.03)	0.02–0.16	*<0.001*
Previous animal husbandry experience	No	*Referent*		
	Yes	1.30 (0.38)	0.72–2.31	0.38
Reference bonus	No	*Referent*		
	Yes	1.45 (0.39)	0.86–2.46	0.16
Attendance special training/conferences	No	*Referent*		
	Yes	1.29 (0.40)	0.71–2.36	0.40

c Statistical significance was declared at *P <* 0.05 and tendencies were defined as 0.05 < *P* ≤ 0.10.

The final multivariable mixed effects ordered logistic regression model for stress levels showed that the odds of experiencing a higher level of stress decreased with increased job satisfaction (Table 3). Respondents with ‘some’ English comprehension when spoken to had higher odds of experiencing a higher level of stress compared to respondents able to understand everything in English, in contrast to respondents understanding ‘a little’ or ‘most’ English at the workplace (Table 3).

**Table 3:** Results from a multivariable mixed effects ordered logistic regression model investigating predictors for stress levels in U.S TN-visa workers^d^.

Variable	Categories	Odds ratio (SE)	95% CI	*P*-value
Position	Farm manager	*Referent*		
	Production manager trainee	0.52 (0.38)	0.13–2.16	0.37
	Team lead	1.18 (0.72)	0.36–3.92	0.78
	Hourly employee	0.54 (0.29)	0.19–2.16	0.25
English comprehension	Very little	*Referent*		
	A little	1.24 (0.45)	0.61–2.54	0.55
	Some	2.33 (0.85)	1.14–4.75	*0.02*
	Most	1.72 (0.63)	0.84–3.53	0.14
Job satisfaction	Very unsatisfied	*Referent*		
	Somewhat unsatisfied	0.13 (0.11)	0.02–0.70	*0.018*
	Neither satisfied nor unsatisfied	0.05 (0.04)	0.01–0.24	*<0.001*
	Somewhat satisfied	0.09 (0.07)	0.02–0.45	*<0.001*
	Very satisfied	0.03 (0.03)	0.01–0.18	*<0.001*
Field of study	Agronomy	*Referent*		
	Animal science	1.83 (0.61)	0.95–3.53	0.07
	Veterinary medicine	1.29 (0.38)	0.72–2.30	0.40
	Biology/ecology	0.77 (0.38)	0.29–2.03	0.59

d Statistical significance was declared at *P <* 0.05 and tendencies were defined as 0.05 < *P* ≤ 0.10.

The final multivariable mixed logistic regression model for “interest in seeking new employer” showed that in-person survey respondents had lower odds of considering changing employer compared to online-survey respondents (Table 4). Respondents currently employed on farms with herd sizes between 2,501 and 5,000 pigs, had approximately 60% lower odds of considering changing employer compared to respondents currently working on farm with fewer than 2,500 pigs (Table 4). Respondents with very high job satisfaction had approximately 90% lower odds of considering changing employer compared to respondents with the lowest job satisfaction level (Table 4). Finally, respondents with previous animal husbandry experience showed a 2.5-fold increase in the odds of considering changing employer compared to respondents with no prior husbandry experience (Table 4).

**Table 4:** Results from a multivariable mixed effects logistic regression model investigating predictors for U.S TN-visa workers considering changing their current employer^e^.

Variable	Categories	Odds ratio (SE)	95% CI	P-value
Survey type	Online	*Referent*		
	In person	0.29 (0.15)	0.11–0.78	*0.014*
Age	18–34	*Referent*		
	35–44	0.84 (0.35)	0.36–1.93	0.67
	45–65	0.37 (0.25)	0.10–1.37	0.14
Herd size	0–2,500	*Referent*		
	2,501–5,000	0.36 (0.18)	0.14–0.95	*0.048*
	>5,000	0.77 (0.38)	0.29–2.02	0.59
Training	Weekly	*Referent*		
	Monthly	0.58 (0.45)	0.12–2.70	0.49
	Yearly	1.22 (1.10)	0.21–7.17	0.83
	Never	1.36 (1.11)	0.28–6.75	0.70
Stress level	Not stressful at all	*Referent*		
	Somewhat stressful	0.54 (0.45)	0.13–2.25	0.32
	Neither stressful nor not stressful	0.35 (0.21)	0.11–1.13	0.92
	A little stressful	0.83 (0.45)	0.29–2.39	0.95
	Very stressful	4.63 (4.17)	0.79–27.0	0.09
Previous animal husbandry experience	No	*Referent*		
	Yes	2.48 (1.03)	1.10–5.60	*0.029*
Job satisfaction	Very unsatisfied	*Referent*		
	Somewhat unsatisfied	2.61 (3.08)	0.26–26.5	0.42
	Neither satisfied nor unsatisfied	1.07 (1.17)	0.13–9.04	0.92
	Somewhat satisfied	1.00 (1.07)	0.13–8.05	0.95
	Very satisfied	0.11 (0.12)	0.01–0.98	*0.048*
Reference bonus	No	*Referent*		
	Yes	0.69 (0.25)	0.34–1.38	0.29

e Statistical significance was declared at *P <* 0.05 and tendencies were defined as 0.05 < *P* ≤ 0.10.

## 4 Discussion

This study explored self-reported job satisfaction, stress levels and interest of changing employment among TN-visa workers in the U.S. swine industry. Most TN-visa workers (72.8%) that participated in the study were satisfied with their current employment while 11.9% described themselves as unsatisfied and 15.3% did not feel strongly in either direction.

The content analysis showed that the main themes of satisfaction were related to positive perceptions of working conditions, compensation and benefits, co-workers, and management. Unsurprisingly, the same themes show up for unsatisfied respondents, but in a different order of importance. While good management was ranked as the fourth most mentioned reason for satisfaction, poor management was the leading theme for dissatisfaction, followed by poor working conditions, poor compensation/benefits, and co-worker conflict. Thus, poor management and poor working conditions outweigh poor compensation and lack of benefits, giving valuable insight that broader systematic changes alongside proper renumeration might be needed to increase retention rates. This result is in line with previous findings suggesting that within the general workforce, compensation has a limited impact on job satisfaction [18].

In-person respondents were more likely to self-report a higher level of job satisfaction compared to online respondents. Despite the assurances of anonymity and data confidentiality, it's possible that in-person respondents were hesitant to provide honest opinions for fear of retaliation or negative consequences and instead felt inclined to provide more socially desirable answers [19], [20]. Moreover, age seemed to have an association with job satisfaction, as respondents older than 35 years of age were more likely to be more satisfied compared to younger respondents. It is possible that job experience and increased English proficiency contributed to the job satisfaction in older respondents. However, the opposite association was observed for respondents with more than 72 months in the TN-visa program. Although not asked in this survey, a longer tenure in the TN-visa program may have exposed participants to more systematic issues leading to lower job satisfaction such as discrimination, unfair treatment by employers [9], [14], lack of promotion opportunities, or being barred from holding high-skilled positions [9], [13].

The respondents ranked competitive salary, and job benefits high for job satisfaction which aligns with previous findings on agricultural worker views [21]. The same study also reported that supervisors are more likely to have a higher degree of job satisfaction and more satisfied with their salary compared to crew members in non-supervisory roles [21]. Low compensation schemes are not uncommon and often directed towards migrant workers resulting in more working hours to reach similar pay while increasing risk of work-related injuries [22], [23]. The risk of workplace injuries is high for migrant workers and may also be compounded by language barriers, working with large animals, agricultural machinery, or lack of healthcare coverage [24], [25], [26], [27], [28]. Almost all respondents reported that they received PTO and a majority were eligible for health insurance, retirement plans and production bonuses. In terms of importance, eligible respondents ranked PTO and healthcare the highest reasons for job satisfaction, followed by production bonuses and retirement plans, while ineligible respondents ranked access to health insurance and retirement plans the highest followed by PTO and production bonuses.

This study also showed that benefit eligibility may vary noticeably between different companies within the same region and for employees within companies. For instance, when comparing nine companies with the highest number of respondents in this study, the average retirement plan and production bonus eligibility was 62.1% and 63.6% but ranged from 0% to 100%, clearly showing differences in offered benefits. Matching benefits to either nearby companies or other positions within the company could be an easily implementable strategy to retain TN-visa workers long-term. The variability in self-reported benefits may possibly reflect differences in participants' awareness, enrollment, or company-specific eligibility requirements or availability. It is our understanding that benefits do not vary substantially by employee position within the companies represented; however, this was not explicitly assessed, as it was outside the scope of the present study. We recognize the likelihood that self-reported information may have created variations based on differences in interpretation or recall. Responses regarding participation in retirement plans possibly explained as a result of hesitation about long-term residency in the U.S. or the portability of retirement funds, influencing individuals' participation in this benefit. In the case of production bonuses, the reported differences across companies are due to availability or eligibility criteria, with some companies requiring specific training, certifications, or experience within certain production stages before employees qualify.

Increased stress levels significantly decreased the likelihood of having high job satisfaction. Although unsurprising due to the inherent close association, specific farm roles may contribute to stress levels. For instance, respondents holding positions as manager-trainees or hourly employees were significantly less likely to have high job stress compared to farm managers. To speculate, trainee positions may induce stress through expectations for rapid learning and increased responsibilities while working with the manager, while hourly workers are more likely to be subjected to tougher working conditions with limited job benefits compared to managerial roles. Increased stress levels have been associated with a plethora of factors such as lack of training, unsafe working conditions, discrimination, or language barriers [29], [30], [31]. Respondents that self-reported themselves as having ‘some’ English proficiency were more likely to be stressed compared to respondents with ‘very little’ English comprehension. This finding aligns with previous studies on Spanish speaking agricultural workers, indicating that miscommunication can lead to a multitude of scenarios affecting both animal and employee welfare and safety [28], [30], [32], [33].However, the same association was not found for respondents categorizing themselves as understanding either ‘a little’ or ‘most’ English. This result is difficult to elucidate and should be investigated further and in more detail in future studies. One hypothesis would be that those with ‘some’ English proficiency might be put in more difficult and stressful positions to act as a ‘bridge’ between non-English speakers and English-only speakers, bringing an added responsibility that is not necessarily compensated in any manner.

In-person respondents were less likely to consider a job change compared to online respondents. As mentioned previously, in-person respondents may be more reluctant to answer truthfully but the diverse geographical distribution of online respondents may have captured workers at more companies with poorer work conditions compared to the ones where surveys were distributed in person. Additionally, respondents taking the online survey may already been discontented at their current workplace and thus more inclined to use the survey as an opportunity to voice issues compared to the scheduled, albeit voluntary, in-person survey at the workplace.

Respondents working with swine herds ranging between 2,501 and 5,000 heads were less likely to consider an employer change compared to respondents working with smaller herds. Although hard to deduce from this study, larger sites are more likely to have competitive salaries and benefits, workload monitoring, and provide job security to a larger extent than smaller operations. However, this was not observed for respondents working on sites with herds above 5,000 head. It is possible that there is a reasonable trade-off between farm size, workload, job security, and salary and benefits, where medium sized farms offer favorable conditions. Furthermore, herds larger than 5,000 head could also be more demanding in regard to workload and expectations, and these may cause additional stress besides potential better compensations. Larger facilities may also contribute to less ‘personal contact’ between employees (due to higher numbers), which may contribute to less satisfaction with the work environment, which could lead to employees seeking other opportunities. This needs to be better investigated in the future.

Finally, respondents with previous animal husbandry experience were more likely to consider changing employers compared to those with no previous husbandry experience. Previously learned and applied husbandry skills may conflict with how animals are cared for at specific farms. Unlearning or adapting to routines or codes of practice at odds with previous experience may result in disagreements with management, team‑leaders or co-workers or workplace frustration. Poor caretaker attitudes can negatively affect human-animal interactions, leading to fearful animals [34], [35], and overall poor husbandry [36], [37], [38], emphasizing the importance of maintaining good job satisfaction and a low-stress work environment for caretakers. To speculate, respondents with prior animal husbandry experience or education may also reflect a subgroup that feels underappreciated or underpaid based on said experience or education, and therefore more likely to seek better opportunities elsewhere.

This study does have limitations. The multi-modal nature of job satisfaction and stress is likely to include factors better captured in form of qualitative interviews thus leaving out nuance that could otherwise better explain some of the observed results [39], [40]. Finally, many TN-visa job sites are in rural areas with limited access to amenities and Hispanic cultural communities. These off-work factors may contribute to workers leaving despite overall good work conditions [12], [41]. It is also important to note that data categorization was done solely considering sample size for each category, and in an attempt to keep as many levels as possible to bring more information to the analysis; but this may not had been the best option because it is difficult to quantify and understand participant interpretation of each level, which can be subjective. In the future, methodological studies can focus on ways to standardize these types of questions to obtain the best data quality.

## 5 One Health implication

The findings of this study align strongly with the One Health framework by illustrating the connection between human wellbeing, animal welfare, the environment, and livestock system sustainability. From the perspective of human health, TN-visa workers in this study reported a wide range of job satisfaction and stress levels; with higher levels of reported job stress being significantly associated with lower levels of job satisfaction and increased tendency to leave employment. These results may pose TN-visa workers at risk, including on-farm injuries, due to language barriers and lack of access to resources. Therefore, by improving working conditions, including those related to environmental conditions, language and communication, and access to benefits may directly enhance worker wellbeing and reduce occupational health risks.

From the workforce and system sustainability perspective, job satisfaction and access to benefits were two major factors contributing to retention of TN-visa workers. Since the need for TN-visa workers has grown because of shortages of workers in the swine industry, the issues related to high turnover are substantial when considering operational stability and success within the industry. This study indicates that by enhancing current management practices and providing better compensation packages and education, it may translate into retaining experienced TN-visa workers and will be critical for both financial sustainability and maintaining knowledge base, as well as maintaining the day-to-day operations of the farms.

These human factors have significant effects on the health and welfare of animals. Previous studies have shown that attitudes, stress levels and training of caretakers affect how humans interact with animals, which in turn affects animal behavior, welfare and productivity [42], [43], [44]. In this study, we looked at how these factors (e.g., stress levels, communication barriers, and previous animal husbandry experience) affected workers and their work-related issues, which indirectly impact how animals are cared for. The high levels of turnover and instability in the workforce can further complicate the care and handling of animals since there will be less consistency in animal handling. This can have negative impacts on animal welfare. Additionally, when the workforce is stable and workers are well trained, they will contribute to better on-farm environment and farm management practices; this is because their labor consistency enables them to stick to protocols, creating less conflict among farm workers. Therefore, improving workers' satisfaction levels and reducing their levels of stress will not just improve the individual worker's wellbeing but also contribute to more sustainable and resilient swine production systems. In summary, our study demonstrates that improving worker wellbeing is not just a worker or management issue, but also a vital part of a One Health approach to livestock production in which the health of humans, animals and the environment are all interconnected.

The following are the supplementary data related to this article.

Supplementary Table 1 Self-reported English language proficiency among U.S. TN-visa workers.

Supplementary Table 2 Distribution (%) of job benefit eligibility among TN-visa workers (N = 216) per company with more than 5 respondents from both in-person and online surveys.

## CRediT authorship contribution statement

**Magnus R. Campler:** Writing – review & editing, Writing – original draft, Formal analysis, Data curation. **Andréia G. Arruda:** Writing – review & editing, Funding acquisition, Formal analysis, Data curation, Conceptualization. **Kara Flaherty:** Writing – review & editing, Data curation. **Talita Resende:** Writing – review & editing, Funding acquisition, Data curation, Conceptualization. **Isaiah Franco:** Writing – review & editing, Formal analysis, Data curation. **Douglas Jackson-Smith:** Writing – review & editing, Formal analysis, Data curation, Conceptualization. **Timothy J. Safranski:** Writing – review & editing, Funding acquisition, Conceptualization. **Magdiel Lopez-Soriano:** Writing – review & editing, Visualization, Project administration, Funding acquisition, Data curation, Conceptualization.

## Funding

This project was sponsored by the 10.13039/100008370National Pork Board (NPB Project RfP-0042 – MU Project 00086920).

## Declaration of competing interest

The authors declare no conflict of interest.

## Data Availability

Data will be made available on request.

## References

[1] Black N.J., Arruda A.G. (2021). Turnover events of animal caretakers and its impact on productivity in swine farms. Prev. Vet. Med..

[2] Lichter D.T., Johnson K.M. (2020). A demographic lifeline? Immigration and Hispanic population growth in rural America. Popul. Res. Policy Rev..

[3] Martin P. (2015).

[4] Orrenius P., Streitfeld D. (2006). TN visas: a stepping stone toward a NAFTA labor market. Southwest. Econ..

[5] Acevedo León N.F., Jaramillo P.L., Gabela C.D., Boren-Alpízar A., Andrukonis A., Schmidt M., McGlone J., Garcia A. (2024). Hispanic worker attitudes toward pig euthanasia on U.S. farms. Front. Vet. Sci..

[6] Hernández-León R., Sandoval E. (2024). The end of Mexico–US migration as we knew it – or back to the future?. Transitions.

[7] National Pork Board (2017). Employee Compensation & HR Practices in Pork Production, 2016–2017 Report. https://www.porkcdn.com/sites/porkorg/library/2012/09/2016-NPB-compensation-survey.pdf.

[8] Ramos A.K., Reynaga D. (2023). The TN visa: the future of foreign workers in livestock production. J. Agromed..

[9] U.S Department of Labor, Employment and Training Administration (2014). National Agricultural Workers Survey, Public Data, 1989–2012. https://www.dol.gov/sites/dolgov/files/ETA/naws/pdfs/NAWS_Research_Report_10.pdf.

[10] Leech S.R., Greenwood E. (2010). Keeping America competitive: a proposal to eliminate the employment-based immigrant visa quota. Alb. Gov’t L. Rev..

[11] National Pork Board (2022). Pork Industry labor study. https://porkcheckoff.org/wp-content/uploads/2022/08/National-Pork-Board-Labor-Study.pdf.

[12] Boessen C., Artz G., Schulz L. (2018).

[13] Mauldin E. (2017). https://cdmigrante.org/wp-content/uploads/2018/01/Coerced-under-NAFTA_-Abuses-of-Migrant-Workers-in-TN-Visa-Program.pdf.

[14] Campler M.R., Pairis-Garcia M.D., Rault J.-L., Coleman G., Arruda A.G. (2018). Caretaker attitudes toward swine euthanasia. Transl. Anim. Sci..

[15] Yarian J., Johnson A.K., Skaar B., Stalder K.J., Pairis-Garcia M.D., Robles I., Arruda A.G., Jass C. (2022).

[16] Lopez-Soriano M., Resende T., Arruda A.G., Campler M.R., Franco I., Flaherty K., Johnson A., Pairis-Garcia M.D., Chatila S., Pieters M., Urriola P., Adams K.L., Begum N., Jackson-Smith D., Safranski T. (2026). Translational Animal Science.

[17] Kleinheksel A., Rockich-Winston N., Tawfik H., Wyatt T.R. (2020). Demystifying content analysis. Am. J. Pharm. Educ..

[18] Judge T.A., Piccolo R.F., Podsakoff N.P., Shaw J.C., Rich B.L. (2010). The relationship between pay and job satisfaction: a meta-analysis of the literature. J. Vocat. Behav..

[19] De Leeuw E.D. (2018).

[20] Williams C.C., Schneider F. (2016). Measuring the Global Shadow Economy.

[21] Hobbs M., Klachky E., Cooper M. (2020). Job satisfaction assessments of agricultural workers help employers improve the work environment and reduce turnover. Calif. Agric..

[22] Dembe A.E., Erickson J.B., Delbos R.G., Banks S.M. (2005). The impact of overtime and long work hours on occupational injuries and illnesses: new evidence from the United States. Occup. Environ. Med..

[23] Orrenius P.M., Zavodny M. (2009). Do immigrants work in riskier jobs?. Demography.

[24] Byler C.G. (2013). Hispanic/Latino fatal occupational injury rates. Monthly Lab. Rev..

[25] Yanar B., Kosny A., Smith P.M. (2018). Occupational health and safety vulnerability of recent immigrants and refugees. Int. J. Environ. Res. Public Health.

[26] Swanton A.R., Young T.L., Peek-Asa C. (2016). Characteristics of fatal agricultural injuries by production type. J. Agric. Saf. Health.

[27] Ramos A.K., Carlo G., Grant K., Trinidad N., Correa A. (2016). Stress, depression, and occupational injury among migrant farmworkers in Nebraska. Safety.

[28] Ramos A.K., Adhikari S., Yoder A.M., Rautiainen R.H. (2021). Occupational injuries among Latino/a immigrant cattle feedyard workers in the central states region of the United States. Int. J. Environ. Res. Public Health.

[29] Furgurson K.F., Quandt S.A., Arcury T.A., Quandt S.A. (2020). Latinx Farmworkers in the Eastern United States.

[30] Arcury T.A., Grzywacz J.G., Sidebottom J., Wiggins M.F. (2013). Overview of immigrant worker occupational health and safety for the agriculture, forestry, and fishing (AgFF) sector in the southeastern United States. Am. J. Ind. Med..

[31] Luksyte A., Spitzmueller C., Rivera-Minaya C.Y. (2014). Factors relating to wellbeing of foreign-born Hispanic workers. J. Manag. Psychol..

[32] Hemsworth P. (1997).

[33] Clouser J.M., Bush A., Gan W., Swanberg J. (2018). Associations of work stress, supervisor unfairness, and supervisor inability to speak Spanish with occupational injury among Latino farmworkers. J. Immigr. Minor. Health.

[34] Breuer K., Hemsworth P.H., Barnett J.L., Matthews L.R., Coleman G.J. (2000). Behavioural response to humans and the productivity of commercial dairy cows. Appl. Anim. Behav. Sci..

[35] Coleman G.J., Hemsworth P.H., Hay M. (1998). Predicting stockperson behaviour towards pigs from attitudinal and job-related variables and empathy. Appl. Anim. Behav. Sci..

[36] Ceballos M.C., Sant’Anna A.C., Boivin X., de O. Costa F., de L. Carvalhal M.V., Paranhos da Costa M.J.R. (2018). Impact of good practices of handling training on beef cattle welfare and stockpeople attitudes and behaviors. Livest. Sci..

[37] Coleman G.J., McGregor M., Hemsworth P.H., Boyce J., Dowling S. (2003). The relationship between beliefs, attitudes and observed behaviours of abattoir personnel in the pig industry. Appl. Anim. Behav. Sci..

[38] Leon A.F., Sanchez J.A., Romero M.H. (2020). Association between attitude and empathy with the quality of human-livestock interactions. Animals.

[39] Goyes D.R., Sandberg S. (2025). Trust, nuance, and care: advantages and challenges of repeat qualitative interviews. Qual. Res..

[40] Knott E., Rao A.H., Summers K., Teeger C. (2022). Interviews in the social sciences. Nat. Rev. Methods Primers.

[41] Arcury T.A., Quandt S.A., Arcury T.A., Quandt S.A. (2020). Latinx Farmworkers in the Eastern United States: Health, Safety, and Justice.

[42] Leon N.F.A., Garcia A., Schroeder K., Boren A., Lamino P. (2022). 6 validation and application of a survey of on-farm worker attitudes towards euthanasia. J. Anim. Sci..

[43] Pol F., Kling-Eveillard F., Champigneulle F., Fresnay E., Ducrocq M., Courboulay V. (2021). Human–animal relationship influences husbandry practices, animal welfare and productivity in pig farming. Animal.

[44] Palacios C., Plaza J., Abecia J.-A. (2021). A high cattle-grazing density alters circadian rhythmicity of temperature, heart rate, and activity as measured by implantable bio-loggers. Front. Physiol..

