# Supplementary material for: Underlying factors influencing job satisfaction and stress among TN-visa workers in the U.S. swine industry
Source: One Health. 2026 Jun 27;23:101500. doi: 10.1016/j.onehlt.2026.101500 (PMC13330621; doi:10.1016/j.onehlt.2026.101500)
Supplement: Supplementary Table 1 — Self-reported English language proficiency among U.S. TN-visa workers. [file mmc1.docx]

**Supplementary table 1.** Self-reported English language proficiency among U.S. TN-visa workers.

|  |  | **N** | **%** |  |  |  |  |
| --- | --- | --- | --- | --- | --- | --- | --- |
|  |  |  |  |  |  |  |  |
| **English comprehension** |  |  |  |  |  |  |  |
| *Very little* |  | 50 | 19.2 |  |  |  |  |
| *A little* |  | 53 | 20.4 |  |  |  |  |
| *Some* |  | 76 | 29.3 |  |  |  |  |
| *Most* |  | 75 | 28.8 |  |  |  |  |
| *Everything* |  | 6 | 2.3 |  |  |  |  |
|  |  |  |  |  |  |  |  |
| **English fluency** |  |  |  |  |  |  |  |
| *Very little* |  | 57 | 21.9 |  |  |  |  |
| *A little* |  | 72 | 27.7 |  |  |  |  |
| *Some* |  | 76 | 29.2 |  |  |  |  |
| *Most* |  | 53 | 20.4 |  |  |  |  |
| *Everything* |  | 2 | 0.8 |  |  |  |  |
|  |  |  |  |  |  |  |  |
|  |  | **Age (%)** | | | |  |  |
| **Overall English proficiency** |  | **18-34** | **35-44** | **45-64** |  |  |  |
| *Basic (few words and phrases)* |  | 66.2 | 67.5 | 48.1 |  |  |  |
| *Intermediate (can hold a conversation)* |  | 28.0 | 28.4 | 48.1 |  |  |  |
| *Advanced (fluent, full conversation with confidence)* |  | 5.8 | 4.1 | 3.8 |  |  |  |
