# Supplementary material for: Underlying factors influencing job satisfaction and stress among TN-visa workers in the U.S. swine industry
Source: One Health. 2026 Jun 27;23:101500. doi: 10.1016/j.onehlt.2026.101500 (PMC13330621; doi:10.1016/j.onehlt.2026.101500)
Supplement: Supplementary Table 2 — Distribution (%) of job benefit eligibility among TN-visa workers (N = 216) per company with more than 5 respondents from both in-person and online surveys. [file mmc2.docx]

**Supplementary table 2.** Distribution (%) of job benefit eligibility among TN-visa workers (N=216) per company with more than 5 respondents from both in-person and online surveys.

| **Company** | **N** | **Healthcare** | **Retirement plan** | **Production bonus** | **Special conference/training** | **PTO*** | **Referral bonus** |
| --- | --- | --- | --- | --- | --- | --- | --- |
| A | 90 | 100.0 | 92.2 | 86.7 | 32.2 | 96.7 | 60.0 |
| B | 29 | 0 | 0 | 0 | 10.3 | 100.0 | 75.9 |
| C | 25 | 100.0 | 36.0 | 68.0 | 32.0 | 92.0 | 36.0 |
| D | 18 | 0 | 33.3 | 66.7 | 11.1 | 100.0 | 22.2 |
| E | 16 | 100.0 | 62.5 | 68.8 | 37.5 | 100.0 | 0 |
| F | 12 | 100.0 | 75.0 | 16.7 | 16.7 | 100.0 | 58.3 |
| G | 11 | 100.0 | 72.7 | 90.9 | 72.7 | 100.0 | 9.1 |
| H | 8 | 100.0 | 87.5 | 75.0 | 37.5 | 100.0 | 50.0 |
| I | 7 | 100.0 | 100.0 | 100.0 | 28.6 | 100.0 | 28.6 |

*Paid time off
